# Monoamine Oxidases, Oxidative Stress, and Altered Mitochondrial Dynamics in Cardiac Ageing

**DOI:** 10.1155/2017/3017947

**Published:** 2017-05-04

**Authors:** Damien Maggiorani, Nicola Manzella, Dale E. Edmondson, Andrea Mattevi, Angelo Parini, Claudia Binda, Jeanne Mialet-Perez

**Affiliations:** ^1^Institut des Maladies Métaboliques et Cardiovasculaires, INSERM, Université de Toulouse, Toulouse, France; ^2^Department of Biochemistry, Emory University School of Medicine, 1510 Clifton Road, Atlanta, GA, USA; ^3^Department of Biology and Biotechnology, University of Pavia, Via Ferrata 1, 27100 Pavia, Italy

## Abstract

The advances in healthcare over the past several decades have resulted in populations now living longer. With this increase in longevity, a wider prevalence of cardiovascular diseases is more common and known to be a major factor in rising healthcare costs. A wealth of scientific evidence has implicated cell senescence as an important component in the etiology of these age-dependent pathologies. A number of studies indicate that an excess of reactive oxygen species (ROS) contributes to trigger and accelerate the cardiac senescence processes, and a new role of monoamine oxidases, MAO-A and MAO-B, is emerging in this context. These mitochondrial enzymes regulate the level of catecholamines and serotonin by catalyzing their oxidative deamination in the heart. MAOs' expression substantially increases with ageing (6-fold MAO-A in the heart and 4-fold MAO-B in neuronal tissue), and their involvement in cardiac diseases is supposedly related to the formation of ROS, via the hydrogen peroxide produced during the substrate degradation. Here, we will review the most recent advances in this field and describe why MAOs could be effective targets in order to prevent age-associated cardiovascular disease.

## 1. Introduction

In 2010, the worldwide population of persons aged > 65  years was estimated to be 532 million, a 33 million increase since 2000, and it is projected to represent 16.7% (1550 million) of the worldwide population by year 2050 [World Health Organization, 2013]. Although the increase in longevity represents a progress per se, it can become a burdening social, economical, and medical problem when it is not associated with the maintenance of the quality of life and the prevention of disability. Several age-related conditions potentially promoting the disabling process are now often described as the “frailty syndrome.” “Frailty” is a clinical state in which patients are at risk for events (i.e., falls, ageing-associated chronic diseases, and hospitalization) increasing the incidence of disability and mortality [1]. In particular, age-associated cardiovascular diseases constantly increase after 55 and are known to be one of the major factors promoting frailty and disability. This notion should be viewed in light of the converging results suggesting that the incidence of heart failure in ageing is related to the acceleration of the intrinsic cardiac senescence program. This phenomenon concerns the cardiomyocytes, the contractile component of the heart [2] as well as the cardiac stroma cells involved in the regulation of cardiac homeostasis [3]. In this review, we will (1) give an update of the most recent developments in cardiac ageing, (2) describe the central role of mitochondrial ROS in cardiac ageing, and (3) discuss the role of monoamine oxidases (MAOs) as potential drivers of cardiac ageing.

## 2. Cardiac Ageing

For cardiomyocytes, ageing is associated with a nonreplicative or postmitotic senescence, which displays a number of pathological and morphological features such as increased sensitivity to apoptosis and necrosis, cellular hypertrophy, alterations in contraction and relaxation, and depressed bioenergetics [4]. The molecular pathways involved in postmitotic senescence of cardiomyocytes are far from being understood and seem to be distinct from replicative senescence, although some key molecular actors are shared. During replicative senescence, the loss of telomeric DNA during erosion of chromosome ends is sensed as a persistent DNA damage signal, which in turn activates the DNA damage response pathway resulting in enhanced expression of cyclin-dependent kinase inhibitors (CDKi) p16^INK4a^ and p53. In the heart, telomere attrition and expression of p16^INK4a^ have been observed in senescent cardiomyocytes and have been linked to hypertrophy in both rodents and humans [5, 6]. However, based on the poor replicative potential of the heart, only a small proportion of cardiomyocytes displays telomeres shortening (about 16%) in aged rat hearts which contrasts with the high percentage of senescent cardiomyocytes positive for p16^INK4a^ [6]. This suggests an alternative mechanism of senescence in the heart which will warrant further investigations. Interestingly, as recently demonstrated by Baker et al., removing the p16^INK4a^-positive senescent cells in adult mice not only increases life span but also protects the heart from cardiac hypertrophy and isoproterenol stress [7]. This study demonstrates for the first time the deleterious role of senescent cells during cardiac ageing. In addition, mice deficient for telomerase display telomeres shortening after four generations (G4) and develop cardiomyopathy characterized by enhanced cardiomyocyte death and cellular hypertrophy [8]. In this study, the authors describe a new link between telomere dysfunction and p53 activation, leading to mitochondrial and metabolic impairment through the repression of the master regulator of mitochondrial biogenesis, PGC-1*α* (peroxisome proliferator-activated receptor gamma, coactivator 1*α*) [8]. Thus, telomere DNA damage and metabolic pathways may trigger intracellular events leading to mitochondrial dysfunction, altered energy maintenance, and finally the ageing process. Age-related mitochondrial alterations consist of increased reactive oxygen species (ROS), impaired oxidative phosphorylation, reduced ATP generation, impaired fatty acid oxidation, and increased mutations in mitochondrial DNA [2]. Interestingly, accelerated ageing associated with a cardiomyopathy was also observed in mice with a defective mitochondrial polymerase *γ*, an enzyme involved in mtDNA replication [9]. Thus, mitochondrial damage and dysfunction appear central in the process of ageing.

In order to prevent the deleterious accumulation of damaged mitochondria, a recycling system allows elimination of mitochondria through the autophagy-lysosome process. However, the efficiency of this pathway declines during ageing contributing to cell death or senescence [10]. In experimental models, the disruption of the autophagy-lysosome pathway through Atg5 (autophagy-related 5) knock-down leads to cardiac dilatation and premature ageing [11]. On the other hand, stimulation of autophagy with pharmacological agents such as resveratrol has gained interest in recent years since it has been demonstrated to protect from cardiac failure [12]. Autophagic degradation of mitochondria involves several specific proteins. One of them, Parkin, is an E3 ubiquitin ligase that rapidly moves from cytosol to the outer membrane of dysfunctional mitochondria where it promotes mitophagy. Interestingly, parkin deficiency in mice led to the progressive accumulation of dysfunctional mitochondria whereas parkin overexpression maintained mitochondrial integrity in the ageing heart [13]. During normal ageing, the authors demonstrated that cytosolic p53 was responsible for impaired parkin translocation to the mitochondria and thus impaired mitophagy. In conclusion, compromised recycling of cytoplasmic materials and mitochondria might constitute another fundamental parameter in the ageing process, especially in long-lived postmitotic cardiac cells with limited regenerative capacities [10].

### 2.1. ROS and Ageing

It is now well understood that different stressor factors including oxidative stress, genotoxic agents, and metabolic dysfunction accelerate cardiomyocyte senescence (SIPS: stress-induced premature senescence), which may predispose individuals to premature ageing and heart failure [14].

Reactive oxygen species play a pivotal role in triggering and accelerating cardiac senescence [15]. It is now generally accepted that the ageing process is to a large extent related to macromolecular damage by ROS, mostly affecting long-lived postmitotic cells such as neurons and cardiac myocytes. Because of the inherent chemical properties of oxygen, both respiratory and nonrespiratory O_2_-involving biological processes inevitably generate by-products, the ROS. These include superoxide (O_2_^.^), hydrogen peroxide (H_2_O_2_), and hydroxyl radicals (OH^.^). The past years have witnessed a tremendous growth in the interest and research activities on this topic. The characteristic feature of ROS is their ability to target multiple cell components, therefore exerting deleterious effects on cell functions. Specific to the cardiomyocytes and chronic heart failure, ROS can directly oxidize proteins involved in contractile activity and consequently impair ventricular function [16–18]. They have been shown to interfere with quality control mechanisms by blocking autophagy in the heart, thus promoting senescence and apoptosis [19]. Moreover, ROS activate signal transduction pathways involved in cardiac hypertrophy and trigger a proapoptotic cascade [20]. They also mediate extracellular matrix accumulation through activation of cardiac fibroblasts, leading to ventricular fibrosis [21, 22]. All these effects are associated with the adverse cardiac remodeling and failure. Consistently, ROS sources are intensively pursued targets for effective pharmacological treatments of these pathological conditions [23–25]. Although different sources contribute to global oxidative stress, the vast majority of cellular ROS (90%) originate from the mitochondrial compartment [26]. According to the mitochondrial variant of the free radical theory of ageing, ROS produced in these organelles attack their constituents, causing mitochondrial dysfunction and DNA damage, leading to further increase in ROS, oxidative damage to lipids and proteins, and decline in cellular and organ function [27]. This central role of mitochondrial ROS has been demonstrated in mice with overexpression of the antioxidant enzyme catalase targeted to mitochondria, which display protection from cardiac ageing [28]. Recently, we identified the mitochondrial enzymes MAOs as a prominent source of ROS.

## 3. MAOs in the Ageing Heart

### 3.1. MAO Isoforms

**Figure 1:**
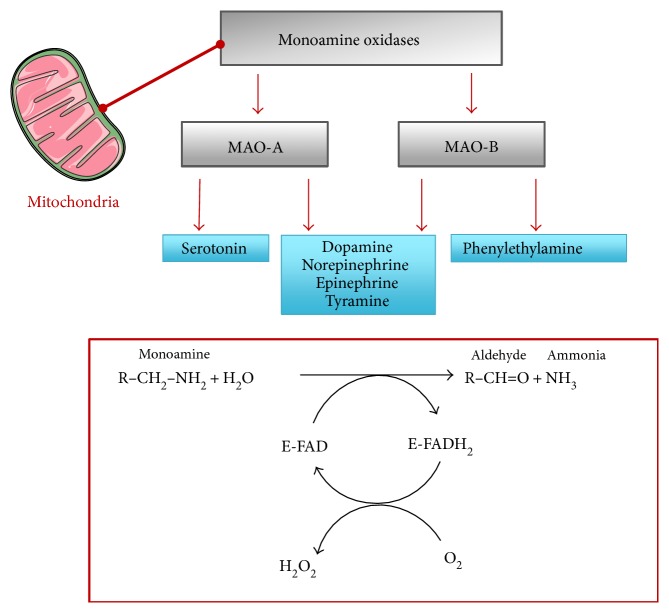
MAO-A and MAO-B share 73% sequence identity and exhibit overlapping but nonidentical specificities in the oxidation of primary amines. Oxidative deamination of monoamines by the flavoenzyme MAO-A proceeds in two steps: in the first step, binding of the monoamine to the enzyme (E) yields an aldehyde and ammonia by reduction of FAD cofactor; in the second step, the oxidized form of the prosthetic group is restored by the binding of oxygen and the concomitant production of hydrogen peroxide (H_2_O_2_). FAD, Flavin adenine dinucleotide.

Besides the respiratory chain, which is considered a major source of mitochondrial ROS in the heart, there is a class of enzymes termed monoamine oxidases, which reside in the outer mitochondrial membrane [29]. Monoamine oxidases A and B (MAO-A and MAO-B) are enzymes of paramount importance in the regulation of catecholamines and other biogenic amines in mammals. They are both expressed at equivalent levels in the human heart but differ significantly in rodents, with MAO-A being the major isoforms in the rat heart whereas MAO-B being expressed predominantly in the mouse heart [30, 31]. Comparison of amino acid sequences show that A and B isoforms have 73% identity in humans. There is also high similarity among species since rodent and human MAO-A display 88% identity. MAOs employ a FAD cofactor to catalyze the oxidative deamination of several monoamines, including not only neurotransmitters (e.g., serotonin, norepinephrine, and dopamine) but also exogenous amines ingested with normal diets (tyramine), generating H_2_O_2_ and the corresponding aldehydes as by-products (Figure 1). MAO-A and MAO-B feature nonidentical but partly overlapping substrate specificities and inhibitor sensitivities. In particular, serotonin is a preferential substrate of MAO-A while catecholamines can be oxidized by both isoforms (Figure 1) [32]. Some substrate overlapping can occur and might become especially relevant in conditions of high substrate concentrations such as heart failure, raising the question of the contribution of each MAO isoform to pathogenesis.

### 3.2. Serotonin and Norepinephrine in Heart Failure

Norepinephrine and serotonin powerfully elicit a variety of biological responses, beyond their roles as neurotransmitters in the central nervous system. In the heart, norepinephrine is released by sympathetic nerve endings whereas serotonin is mainly produced by intestinal enterochromaffin cells and endothelial cells. This so-called peripheral serotonin is stored in the platelets and is released upon platelet-activating processes (i.e., hemostasis or pathological thrombosis). Alternatively, serotonin can also be produced by coronary endothelial cells and therefore may regulate cardiac function independently of platelet activation [33]. The increase in sympathetic nervous system (SNS) activity is typical of chronic heart failure (HF) and is characterized by norepinephrine spillover and decreased neuronal uptake [34]. Physiological ageing is also characterized by SNS dysfunction as shown by the increase in circulating catecholamine levels in old compared to adult individuals [35]. Concerning serotonin, a correlation was found between plasmatic serotonin and the degree of hypertrophy in aortic stenosis patients [36]. In another study, higher serotonin levels were associated with worse HF symptoms and systolic dysfunction [37, 38]. Therefore, the increase in norepinephrine and serotonin levels could participate in cardiovascular dysfunction and may explain the age-associated increase in cardiovascular morbidity and mortality [39]. Indeed, it is now well established that both of these biogenic amines are involved in adverse cardiac remodeling through cardiomyocyte hypertrophy, apoptosis, and necrosis [40–42], ultimately leading to heart failure. The hypertrophic and profibrotic activities of noradrenaline and serotonin may also be particularly relevant in a recently described form of ventricular dysfunction associated with ageing (heart failure with preserved ejection fraction, HFpEF) [43, 44].

### 3.3. MAOs as Relevant Sources of ROS in Age-Associated Cardiac Diseases

**Figure 2:**
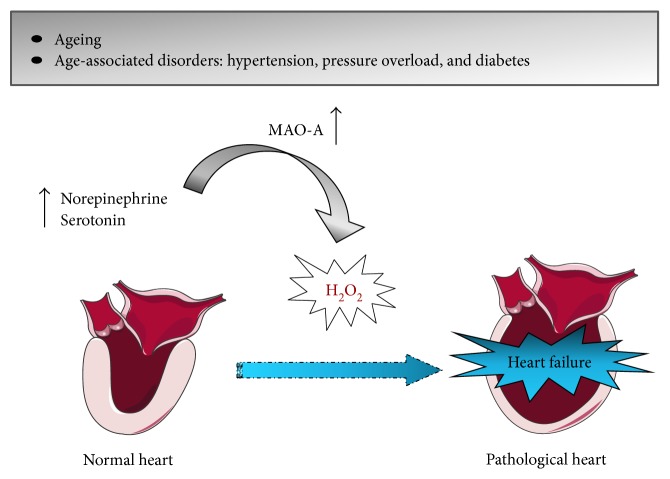
Putative role of MAO-A in heart failure. Ageing- and age-associated disorders show increased MAO-A expression and enhanced release of norepinephrine and serotonin. As a consequence, MAO-A activity is elevated and produces more H_2_O_2_ that in turn, contributes to oxidative stress and heart failure.

Recently, MAO-A and MOA-B were identified as major sources of H_2_O_2_ in the heart that participate in the onset and progression of cardiac injury [30]. Although the role of each isozyme remains to be investigated, it is well known that MAOs' expression and their abilities to produce ROS increase with age [45] and are pronounced in age-associated chronic diseases (i.e., hypertension, pressure overload, and diabetes) [42, 46, 47] (Figure 2).

In light of the established roles of ROS in heart diseases and the pharmacological effects elicited by MAO-inhibitor drugs, these mitochondrial enzymes are now becoming actively investigated as potential targets for the treatment of cardiac dysfunction and ageing. The fundamental discovery is that MAO-A overactivity elicits mitochondrial damage and myocardial degeneration in rodent models of pressure overload or diabetes, which can be effectively prevented by using MAO-inhibiting drugs [42, 48, 49]. This concept becomes even more relevant in light of the well-documented tissue-specific increases in MAO-A and MAO-B levels with age. MAO-B increases 3-4-fold in neuronal tissue (including the brain) which has been shown in animal models to lead to Parkinson-type syndromes [50]. Most significant, MAO-A levels have been shown to increase ~6-fold in the ageing heart, a phenomenon proposed to specifically enhance the effects exerted by factors and conditions that trigger cardiac damage [45]. Moreover, a very recent clinical study showed a correlation between MAO levels and postoperative atrial fibrillation, a cardiac arrhythmia often associated with ageing [51]. These findings and observations provide the framework for the mostly unexplored functions of MAOs in the biology of the ageing heart and associated pathological conditions.

### 3.4. Mechanisms of Action of MAOs in the Heart

**Figure 3:**
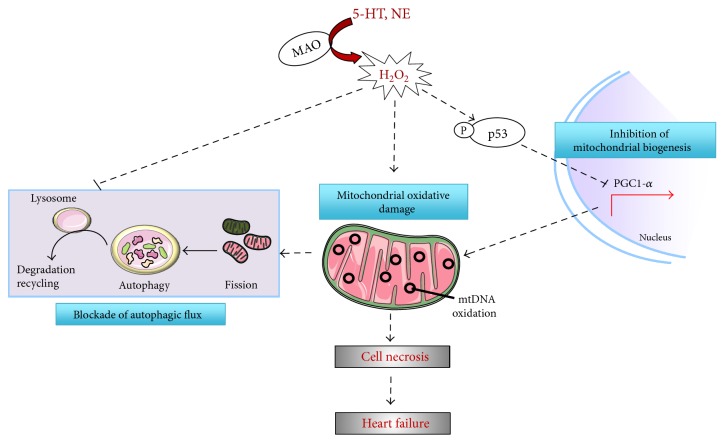
Deleterious effect of MAO-A on mitochondrial damage, cardiomyocyte death, and heart failure. MAO-A-generated oxidative stress triggers p53 activation leading to the downregulation of peroxisome proliferator-activated receptor-gamma coactivator-1*α* (PGC-1*α*), a master regulator of mitochondrial biogenesis. On the other hand, MAO-A-generated oxidative stress impairs lysosome function and acidification leading to autophagic flux blockade and altered mitochondrial quality control.

As mentioned above, MAO-A activities are enhanced in several models of heart failure as well as in the ageing rat heart. In order to investigate the consequences of increased MAO-A activity in heart failure and ageing, Villeneuve and colleagues recently developed in vitro and in vivo models of MAO-A overexpression [42]. In vivo, cardiac-specific overexpression of MAO-A in young mice led to decreased levels of bioamines (norepinephrine and serotonin) together with increased concentrations of the aldehyde metabolites generated by the MAO-catalyzed amine oxidation [42]. At the same moment, mice with cardiac-selective MAO-A overexpression (Tg-MAOA) displayed enhanced levels of H_2_O_2_ in the heart and oxidation of mitochondrial DNA, together with mitochondrial ultrastructural defects. Gene expression analysis by microarray emphasized depressed energy metabolism in Tg-MAOA hearts accompanied by downregulation of the PGC-1*α* pathway involved in mitochondrial biogenesis [42]. Consequently, Tg-MAOA displayed progressive cardiomyocyte necrosis leading to premature death by heart failure at about 9 months of age. In vitro, transduction of cardiomyocytes with a MAO-A adenovirus in the presence of tyramine reproduced mitochondrial damage, diminished ATP production, and decreased PGC-1*α* expression and necrosis through ROS generation. Most interestingly, the activation of p53 by MAO-A was responsible for mitochondrial damage, PGC1*α* downregulation, and cardiomyocyte necrosis (Figure 3).

The autophagy-lysosome pathway is an important mechanism of quality control in the heart for damaged proteins and organelles (mitochondria), but its efficiency decreases during ageing or heart failure [52, 53]. The role of ROS in the regulation of autophagy has been extensively shown. However, the signalling mechanisms through which ROS modulate autophagy in a regulated manner have only been minimally clarified. A recent study demonstrated for the first time that MAO-A activation, through ROS generation, acted as a negative regulator of the autophagy-lysosome pathway [54]. Santin and colleagues found that persistent activation of MAO-A led to the progressive accumulation of LC3-positive autophagosomes, p62, ubiquitylated proteins, and damaged mitochondria. Blockade of autophagic flux was due to impaired lysosomal acidification and function and impaired lysosomal biogenesis through inhibition of the transcription factor EB (TFEB), a master regulator of autophagy-lysosome pathway. Interestingly, gene therapy with cardiomyocyte-driven TFEB adeno-associated vector rescued autophagic dysfunction, cardiac remodeling, and heart failure in MAO-A-overexpressing mice [54] (Figure 3). In conclusion, ROS derived from MAO-A could alter lysosomal function causing a defect in quality control mechanisms. Although most of the changes associated with MAO-A activation, such as mitochondrial damage, p53 activation, PGC-1*α* downregulation, and autophagy blockade mimic accelerated cardiac ageing, additional confirmations will be needed to correlate MAO-A with cardiomyocyte senescence. It is noteworthy that MAO-A-dependent intracellular cascade and mitochondria dysfunction described above are similar to those observed in mouse models with telomeric shortening decribed by Sahin et al. [8]. Therefore, MAO-A seems to be an important intracellular source of ROS triggering premature cardiomyocyte senescence.

Evidence exists also for a role of MAO-B in age-related heart disease. For instance, it was recently shown that genetic deletion of MAO-B protected against oxidative stress, apoptosis, and ventricular dysfunction in a model of pressure overload [55]. Interestingly, the authors demonstrated a direct link between MAO activation and ROS formation inside the mitochondria, which was responsible for the loss of mitochondrial potential in vitro. They analyzed ROS accumulation in a spatiotemporal manner using a redox fluorescent probe targeted specifically to the mitochondrial or cytosolic compartment, and they observed that following MAO-B activation, H_2_O_2_ levels increased much earlier at the mitochondrial level (10 min) than in the cytosol (30 min). Interestingly, this observation demonstrates that H_2_O_2_, which is generated at the outer mitochondrial membrane, can rapidly accumulate inside the matrix and act locally on mitochondrial targets. Indeed, a loss of mitochondrial membrane potential has been described after MAO activation, but also an oxidation of mtDNA, an alteration of mitochondrial ultrastructure and an impairment of respiratory chain [30].

Besides ROS, aldehydes produced during the metabolism of dopamine by MAO-B were recently shown to participate in mitochondrial dysfunction in cardiac cells [55]. In the heart, aldehydes are cleared by aldehyde dehydrogenase 2 (ALDH2), which is the most abundant isoform in this tissue. In vitro, genetic inactivation of ALDH2 with siRNA promoted dopamine-induced accumulation of aldehydes through MAO-B and alteration of mitochondrial membrane potential [55]. In vivo, a recent study demonstrated that ALDH2 deficiency in mice precipitated cardiac ageing with aldehyde overload, accelerated senescence, and impaired autophagic flux [56]. Altogether, those studies support a role for aldehydes in cardiac ageing. However, whether MAO-A activation also produces toxic aldehydes targeting mitochondrial function is still under consideration.

## 4. Conclusion

In the past years, a number of studies have uncovered that MAOs' activation and ROS generation can drive mitochondrial damage and myocardial degeneration. Since altered mitochondria dynamic and function are now considered as major determinants of onset and acceleration of cardiac senescence, the importance of MAOs in these processes and heart failure becomes particularly relevant. Especially with reference to MAO-A, since the p53/PGC1*α* mitochondrial dysfunction axis has been identified as a major pathway involved in postmitotic senescence, this enzyme may constitute an important factor during cardiac ageing that can be a target for drugs exerting cardioprotective actions. Future studies will be needed to provide a more clear understanding of the roles of MAO-A and MAO-B in the cellular and molecular mechanisms linking biogenic amine metabolism and ROS generation to accelerated cardiovascular disease progression in ageing. Interestingly, MAOs could constitute effective drug targets for the treatment of cardiac degeneration and disease.

## References

[1] Ahmed N., Mandel R., Fain M. J. (2007). Frailty: an emerging geriatric syndrome. *The American Journal of Medicine*.

[2] Moslehi J., DePinho R. A., Sahin E. (2012). Telomeres and mitochondria in the aging heart. *Circulation Research*.

[3] Cesselli D., Beltrami A. P., D'Aurizio F. (2011). Effects of age and heart failure on human cardiac stem cell function. *The American Journal of Pathology*.

[4] Sheydina A., Riordon D. R., Boheler K. R. (2011). Molecular mechanisms of cardiomyocyte aging. *Clinical Science (London, England)*.

[5] Chimenti C., Kajstura J., Torella D. (2003). Senescence and death of primitive cells and myocytes lead to premature cardiac aging and heart failure. *Circulation Research*.

[6] Kajstura J., Pertoldi B., Leri A. (2000). Telomere shortening is an in vivo marker of myocyte replication and aging. *The American Journal of Pathology*.

[7] Baker D. J., Childs B. G., Durik M. (2016). Naturally occurring p16(Ink4a)-positive cells shorten healthy lifespan. *Nature*.

[8] Sahin E., Colla S., Liesa M. (2011). Telomere dysfunction induces metabolic and mitochondrial compromise. *Nature*.

[9] Trifunovic A., Wredenberg A., Falkenberg M. (2004). Premature ageing in mice expressing defective mitochondrial DNA polymerase. *Nature*.

[10] Linton P. J., Gurney M., Sengstock D., Mentzer R. M., Gottlieb R. A. (2014). This old heart: cardiac aging and autophagy. *Journal of Molecular and Cellular Cardiology*.

[11] Taneike M., Yamaguchi O., Nakai A. (2010). Inhibition of autophagy in the heart induces age-related cardiomyopathy. *Autophagy*.

[12] Kanamori H., Takemura G., Goto K. (2013). Resveratrol reverses remodeling in hearts with large, old myocardial infarctions through enhanced autophagy-activating AMP kinase pathway. *The American Journal of Pathology*.

[13] Hoshino A., Mita Y., Okawa Y. (2013). Cytosolic p53 inhibits Parkin-mediated mitophagy and promotes mitochondrial dysfunction in the mouse heart. *Nature Communications*.

[14] Serrano M., Blasco M. A. (2001). Putting the stress on senescence. *Current Opinion in Cell Biology*.

[15] Davalli P., Mitic T., Caporali A., Lauriola A., D'Arca D. (2016). ROS, cell senescence, and novel molecular mechanisms in aging and age-related diseases. *Oxidative Medicine and Cellular Longevity*.

[16] Steinberg S. F. (2013). Oxidative stress and sarcomeric proteins. *Circulation Research*.

[17] Li Q., Su D., O'Rourke B., Pogwizd S. M., Zhou L. (2014). Mitochondria-derived ROS bursts disturb calcium cycling and induce abnormal automaticity in guinea pig cardiomyocyte: a theoretical study. *American Journal of Physiology. Heart and Circulatory Physiology*.

[18] Kurokawa S., Niwano S., Niwano H. (2014). Cardiomyocyte-derived mitochondrial superoxide causes myocardial electrical remodeling by downregulating potassium channels and related molecules. *Circulation Journal*.

[19] Terman A., Kurz T., Navratil M., Arriaga E. A., Brunk U. T. (2010). Mitochondrial turnover and aging of long-lived postmitotic cells: the mitochondrial-lysosomal axis theory of aging. *Antioxidants & Redox Signaling*.

[20] Sugden P. H., Clerk A. (2006). Oxidative stress and growth-regulating intracellular signaling pathways in cardiac myocytes. *Antioxidants & Redox Signaling*.

[21] Nabeebaccus A., Zhang M., Shah A. M. (2011). NADPH oxidases and cardiac remodelling. *Heart Failure Reviews*.

[22] Lijnen P. J., van Pelt J. F., Fagard R. H. (2012). Stimulation of reactive oxygen species and collagen synthesis by angiotensin II in cardiac fibroblasts. *Cardiovascular Therapeutics*.

[23] Wu X. Y., Luo A. Y., Zhou Y. R., Ren J. H. (2014). N-acetylcysteine reduces oxidative stress, nuclear factorkappaB activity and cardiomyocyte apoptosis in heart failure. *Molecular Medicine Reports*.

[24] Schwarzer M., Osterholt M., Lunkenbein A., Schrepper A., Amorim P., Doenst T. (2014). Mitochondrial reactive oxygen species production and respiratory complex activity in rats with pressure overload-induced heart failure. *The Journal of Physiology*.

[25] Rautiainen S., Levitan E. B., Mittleman M. A., Wolk A. (2013). Total antioxidant capacity of diet and risk of heart failure: a population-based prospective cohort of women. *The American Journal of Medicine*.

[26] Tatarkova Z., Kuka S., Racay P. (2011). Effects of aging on activities of mitochondrial electron transport chain complexes and oxidative damage in rat heart. *Physiological Research*.

[27] Ziegler D. V., Wiley C. D., Velarde M. C. (2015). Mitochondrial effectors of cellular senescence: beyond the free radical theory of aging. *Aging Cell*.

[28] Dai D. F., Santana L. F., Vermulst M. (2009). Overexpression of catalase targeted to mitochondria attenuates murine cardiac aging. *Circulation*.

[29] Edmondson D. E., Binda C., Wang J., Upadhyay A. K., Mattevi A. (2009). Molecular and mechanistic properties of the membrane-bound mitochondrial monoamine oxidases. *Biochemistry*.

[30] Kaludercic N., Mialet-Perez J., Paolocci N., Parini A., Di Lisa F. (2014). Monoamine oxidases as sources of oxidants in the heart. *Journal of Molecular and Cellular Cardiology*.

[31] Sivasubramaniam S. D., Finch C. C., Rodriguez M. J., Mahy N., Billett E. E. (2003). A comparative study of the expression of monoamine oxidase-A and -B mRNA and protein in non-CNS human tissues. *Cell and Tissue Research*.

[32] Youdim M. B., Edmondson D., Tipton K. F. (2006). The therapeutic potential of monoamine oxidase inhibitors. *Nature Reviews. Neuroscience*.

[33] Rouzaud-Laborde C., Hanoun N., Baysal I. (2012). Role of endothelial AADC in cardiac synthesis of serotonin and nitrates accumulation. *PloS One*.

[34] Floras J. S. (2009). Sympathetic nervous system activation in human heart failure: clinical implications of an updated model. *Journal of the American College of Cardiology*.

[35] Esler M. D., Turner A. G., Kaye D. M. (1995). Aging effects on human sympathetic neuronal function. *The American Journal of Physiology*.

[36] Rouzaud-Laborde C., Delmas C., Pizzinat N. (2015). Platelet activation and arterial peripheral serotonin turnover in cardiac remodeling associated to aortic stenosis. *American Journal of Hematology*.

[37] Nigmatullina R. R., Kirillova V. V., Jourjikiya R. K. (2009). Disrupted serotonergic and sympathoadrenal systems in patients with chronic heart failure may serve as new therapeutic targets and novel biomarkers to assess severity, progression and response to treatment. *Cardiology*.

[38] Selim A. M., Sarswat N., Kelesidis I., Iqbal M., Chandra R., Zolty R. (2016). Plasma serotonin in heart failure: possible marker and potential treatment target. *Heart, Lung & Circulation*.

[39] Rich M. W. (2001). Heart failure in the 21st century: a cardiogeriatric syndrome. *The Journals of Gerontology. Series a, Biological Sciences and Medical Sciences*.

[40] Mialet-Perez J., D'Angelo R., Villeneuve C. (2012). Serotonin 5-HT2A receptor-mediated hypertrophy is negatively regulated by caveolin-3 in cardiomyoblasts and neonatal cardiomyocytes. *Journal of Molecular and Cellular Cardiology*.

[41] Fu Y. C., Chi C. S., Yin S. C., Hwang B., Chiu Y. T., Hsu S. L. (2004). Norepinephrine induces apoptosis in neonatal rat cardiomyocytes through a reactive oxygen species-TNF alpha-caspase signaling pathway. *Cardiovascular Research*.

[42] Villeneuve C., Guilbeau-Frugier C., Sicard P. (2013). p53-PGC-1alpha pathway mediates oxidative mitochondrial damage and cardiomyocyte necrosis induced by monoamine oxidase-A upregulation: role in chronic left ventricular dysfunction in mice. *Antioxidants & Redox Signaling*.

[43] De Keulenaer G. W., Brutsaert D. L. (2011). Systolic and diastolic heart failure are overlapping phenotypes within the heart failure spectrum. *Circulation*.

[44] Loffredo F. S., Nikolova A. P., Pancoast J. R., Lee R. T. (2014). Heart failure with preserved ejection fraction: molecular pathways of the aging myocardium. *Circulation Research*.

[45] Maurel A., Hernandez C., Kunduzova O. (2003). Age-dependent increase in hydrogen peroxide production by cardiac monoamine oxidase A in rats. *American Journal of Physiology. Heart and Circulatory Physiology*.

[46] Pino R., Failli P., Mazzetti L., Buffoni F. (1997). Monoamine oxidase and semicarbazide-sensitive amine oxidase activities in isolated cardiomyocytes of spontaneously hypertensive rats. *Biochemical and Molecular Medicine*.

[47] Duicu O. M., Lighezan R., Sturza A. (2016). Assessment of mitochondrial dysfunction and monoamine oxidase contribution to oxidative stress in human diabetic hearts. *Oxidative Medicine and Cellular Longevity*.

[48] Kaludercic N., Takimoto E., Nagayama T. (2010). Monoamine oxidase A-mediated enhanced catabolism of norepinephrine contributes to adverse remodeling and pump failure in hearts with pressure overload. *Circulation Research*.

[49] Umbarkar P., Singh S., Arkat S., Bodhankar S. L., Lohidasan S., Sitasawad S. L. (2015). Monoamine oxidase-A is an important source of oxidative stress and promotes cardiac dysfunction, apoptosis, and fibrosis in diabetic cardiomyopathy. *Free Radical Biology & Medicine*.

[50] Mallajosyula J. K., Kaur D., Chinta S. J. (2008). MAO-B elevation in mouse brain astrocytes results in Parkinson’s pathology. *PloS One*.

[51] Anderson E. J., Efird J. T., Davies S. W. (2014). Monoamine oxidase is a major determinant of redox balance in human atrial myocardium and is associated with postoperative atrial fibrillation. *Journal of the American Heart Association*.

[52] Marzetti E., Csiszar A., Dutta D., Balagopal G., Calvani R., Leeuwenburgh C. (2013). Role of mitochondrial dysfunction and altered autophagy in cardiovascular aging and disease: from mechanisms to therapeutics. *American Journal of Physiology. Heart and Circulatory Physiology*.

[53] Orogo A. M., Gustafsson A. B. (2015). Therapeutic targeting of autophagy: potential and concerns in treating cardiovascular disease. *Circulation Research*.

[54] Santin Y., Sicard P., Vigneron F. (2016). Oxidative stress by monoamine oxidase-A impairs transcription factor EB activation and autophagosome clearance, leading to cardiomyocyte necrosis and heart failure. *Antioxidants & Redox Signaling*.

[55] Kaludercic N., Carpi A., Nagayama T. (2014). Monoamine oxidase B prompts mitochondrial and cardiac dysfunction in pressure overloaded hearts. *Antioxidants & Redox Signaling*.

[56] Wu B., Yu L., Wang Y. (2016). Aldehyde dehydrogenase 2 activation in aged heart improves the autophagy by reducing the carbonyl modification on SIRT1. *Oncotarget*.

